# ﻿A new species of feather-tailed leaf-toed gecko, *Kolekanos* Heinicke, Daza, Greenbaum, Jackman, Bauer, 2014 (Squamata, Gekkonidae) from the poorly explored savannah of western Angola

**DOI:** 10.3897/zookeys.1127.84942

**Published:** 2022-11-02

**Authors:** Javier Lobón-Rovira, Werner Conradie, Ninda L. Baptista, Pedro Vaz Pinto

**Affiliations:** 1 CIBIO, Centro de Investigação em Biodiversidade e Recursos Genéticos, InBIO Laboratório Associado, Campus de Vairão, Universidade do Porto, 4485-661 Vairão, Portugal; 2 Departamento de Biologia, Faculdade de Ciências, Universidade do Porto, 4099-002 Porto, Portugal; 3 BIOPOLIS Program in Genomics, Biodiversity and Land Planning, CIBIO, Campus de Vairão, 4485-661 Vairão, Portugal; 4 Port Elizabeth Museum, P.O. Box 13147, Humewood 6013, South Africa; 5 Department of Nature Conservation Management, Natural Resource Science and Management Cluster, Faculty of Science, George Campus, Nelson Mandela University, George, South Africa; 6 Instituto Superior de Ciências da Educação da Huíla (ISCED-Huíla), Rua Sarmento Rodrigues, Lubango, Angola; 7 Fundação Kissama, Rua 60 Casa 560, Lar do Patriota, Luanda, Angola; 8 TwinLab CIBIO/ISCED, Instituto Superior de Ciências da Educação da Huíla, Lubango, Angola

**Keywords:** Biodiversity, ct-scan, herpetology, osteology, Reptilia, taxonomy

## Abstract

We here describe a new species of feather-tailed leaf-toed gecko, *Kolekanos*, from southern Benguela Province, Angola, based on morphological and osteological evidence, supported by phylogenetic analysis of mitochondrial data. The new species adds to the rapidly growing and newly-recognised endemic biodiversity of Angola, doubling the number of *Kolekanos* species, breaking the pattern observed within other closely-related African members of a clade of circum-Indian Ocean leaf-toed geckos – *Ramigekko*, *Cryptactites* and *Afrogecko* – all of which are presently monotypic. The new species is easily distinguished from *K.plumicaudus*, based on spine-like (as opposed to feather-like) scales on the margins of the original tail. Phylogenetic analyses also recovered the new taxon as monophyletic, with a well-supported sister relationship to *K.plumicaudus*, from which it differs by a substantial 24.1% NADH-dehydrogenase subunit 2 mitochondrial gene uncorrected p-distance.

## ﻿Introduction

African leaf-toed geckos are among the most ancient and taxonomically problematic Gekkonidae groups in Africa (Lobón-Rovira et al. 2022a). Not surprisingly, these geckos have been the focus of several studies (Underwood 1954; Kluge 1983; Bauer et al. 1997; Bauer and Menegon 2006; Rocha et al. 2011; Gamble et al. 2012; Heinicke et al. 2014; Lobón-Rovira et al. 2022a), being partially resolved into three main groups: a circum-Indian Ocean group, an Afro-Malagasy group, and *Urocotyledon* spp. (see Heinicke et al. 2014; Lobón-Rovira et al. 2022a). However, the relationship within each of these groups still remains unresolved in most cases, with poorly-supported deep phylogenetic nodes and several species still excluded from phylogenetic analysis, due to the lack of fresh genetic material or access to new technologies to obtain DNA from formalin-fixed specimens (Lobón-Rovira et al. 2022a, b).

Circum-Indian Ocean leaf-toed geckos, understood as a monophyletic group that attained their current geographic distributions to reflect the landmasses distributed around the Indian Ocean during the Eocene (~40 mya) (Heinicke et al. 2014), have until recently been considered as a group that includes four genera from mainland Africa (*Afrogecko* [2 spp.], *Ramigekko* [1 sp.], *Cryptactites* [1 sp.] and *Kolekanos* [1 sp.]), *Christinus* from Australia and *Matoatoa* from Madagascar (Heinicke et al. 2014). However, a recent phylogenetic analysis that includes, for the first time, material of *Afrogeckoansorgii*, has demonstrated a paraphyletic status of this species, being consequently described as a new genus (*Bauerius*) as separate clade to the circum-Indian Ocean leaf-toed geckos and rendering the four mainland Africa circum-Indian Ocean leaf-toed geckos as monotypic genera (Lobón-Rovira et al. 2022a). Nevertheless, phylogenetic analysis (Heinicke et al. 2014) also suggested cryptic diversification within *Afrogeckoporphyreus* that requires further investigation.

This new paradigm for circum-Indian leaf-toed geckos has only been addressed thanks to the rapid growth of new molecular techniques in the last two decades and the intensive surveys in previously poorly or unexplored regions in Africa, like Angola (Vaz Pinto et al. 2019, 2021; Lobón-Rovira et al. 2022a).

Access to newly-collected material from these regions has brought new opportunities to understand the evolutionary patterns of African herpetofauna, especially African gekkonids. This is particularly noteworthy in terms of the remarkable increase in knowledge of Angolan herpetofauna, with the description of 34 new species (Conradie et al. 2012a, b, 2013, 2020a, 2022a; Stanley et al. 2016; Ceríaco et al. 2018a, 2020a, b, c, 2021; Branch et al. 2019a, 2021; Marques et al. 2019a, b, 2020, 2022a, b; Hallermann et al. 2020; Nielsen et al. 2020; Baptista et al. 2021; Lobón-Rovira et al. 2021; Parrinha et al. 2021; Wagner et al. 2021) and several new country records (Branch and Conradie 2013; Conradie and Bourquin 2013; Ernst et al. 2014, 2015; Branch et al. 2019b; Conradie et al. 2020b, 2021; Lobón-Rovira et al. 2022c) in the last decade. This increase has been especially evident within gekkonids, where the number of taxa has risen to over 45 recognised species for the country (Marques et al. 2020; Ceríaco et al. 2020a, b; Branch et al. 2021; Lobón-Rovira et al. 2021, 2022c; Conradie et al. 2022b) including two endemic leaf-toed gecko genera, *Kolekanos* (Heinicke et al. 2004) and *Bauerius* (Lobón-Rovira et al. 2022a).

Angolan leaf-toed geckos had previously been considered as members of *Afrogecko* Bauer, Good & Branch, 1997, represented by two species, *A.plumicaudus* Haacke, 2008 and *A.ansorgii* (Boulenger 1902). Until recent studies, both species were poorly known and with very restricted geographical distribution in south-western Angola (Haacke 2008; Agarwal et al. 2017; Marques et al. 2018; Vaz Pinto et al. 2019, 2021). With the availability of new material, both species were subsequently assigned to separate monotypic genera. Heinicke et al. (2014) erected *Kolekanos* to accommodate *A.plumicaudus*, while Lobón-Rovira et al. (2022a) created *Bauerius* to accommodate *A.ansorgii*. Furthermore, the known distribution of these two species have been extended over 200 km north- and southwards (Vaz Pinto et al. 2021) and 300 km northwards (Lobón-Rovira et al. 2022a), respectively. The range extension was especially remarkable for *K.plumicaudus*, which is now known to be present from sea level to over 2000 m a.s.l. and covering different ecological zones in south-western Angola (Vaz Pinto et al. 2021).

Scientific studies have been increasing in Angola in recent years, following a long civil war that prevented fieldwork in this region of Africa for several decades until the early 2000s (Huntley and Ferrand 2019). The improved political stability and strengthening of local institutions have motivated further surveys in the coastal regions of Angola, amongst others, with the aim of assessing the distribution of these poorly-known and emblematic taxa. New material of *Kolekanos*, collected well outside its known distributional range (~180 km north from the northernmost record of *K.plumicaudus*), prompted the current study aiming to investigate the potential diversification within this poorly-known genus. Due to the relevance of this group to understand the evolutionary history of African leaf-toed geckos, we herein also provide an updated phylogenetic hypothesis of the circum-Indian Ocean leaf-toed geckos with newly-collected material of *Kolekanos* from Angola to shed light into the taxonomic, distribution and conservation status of this taxon.

## ﻿Materials and methods

### ﻿Sampling

*Kolekanos* specimens and tissue samples have been collected from Namibe Province, Angola, since 2009 (Vaz Pinto et al. 2021). In August 2021, a new population was detected in southern Benguela Province (~180 km north from the northernmost record of *K.plumicaudus*) and, subsequently, nine specimens were collected from two different sites (Table 1). Specimens collected as vouchers were euthanised with injection of tricaine methanesulphonate (MS222) (Conroy et al. 2009). After euthanasia, specimens were fixed in 10% formalin, after which they were transferred to 70% ethanol for long-term storage in the Museo Nacional de Ciencias Naturales (**MNCN**), Spain and Fundação Kissama (FKH), Angola. For molecular analyses, liver samples were collected prior to formalin fixing and stored in 95–99% ethanol. For each specimen/sample collected, its location was recorded using a handheld GPS, in the WGS84 coordinate system.

### ﻿Molecular data

A mitochondrial gene NADH-dehydrogenase subunit 2 (ND2, 1041 bp) was used, comprising information from nine individuals of *Kolekanos* from the new northern records, to generate data for phylogenetic analysis to explore phylogenetic relationships amongst *Kolekanos* (Table 1). DNA was extracted using EasySpin Genomic DNA Tissue Kit (Citomed, Portugal), following the manufacturer’s protocols. PCR amplifications were performed using the following primers (L4437 and H5540; Macey et al. 1997) and concentrations (5 μl QIAGEN PCR MasterMix, 0.4 μl each primer, 3.2 μl H_2_O and 2 μl DNA (DNA elution were adjusted to extraction results). PCR reactions were adjusted following: initial denaturing step at 95 °C for 15 min, followed by 5 cycles of 95 °C for 30 s, 64 °C for 20 s and 72 °C for 60 s (decreasing annealing temperature by -0.5 °C/cycle), followed by 35 cycles of 95 °C for 30 s, 64 °C for 20 s and 72 °C for 60 s, with a final extension at 60 °C for 10 min. For phylogenetic comparisons, we combined the newly-generated ND2 sequences with previously published sequences from Lobón-Rovira et al. (2022a) and Heinicke et al. (2014), deposited in GenBank (http://www.ncbi.nlm.nih.gov/genbank/). The final dataset consisted of our newly-sequenced material and 204 additional sequences, representing a total of 45 different Gekkonidae genera. As outgroup, we used four members of the genus *Phyllodactylus* (family Phyllodactylidae), representatives of the sister family to the family Gekkonidae (Pyron et al. 2013). All sequences were checked and edited using GENEIOUS Prime v.2021.1.1 (http://www.geneious.com/) and aligned using the MUSCLE plugin for GENEIOUS.

**Table 1.** Detailed collection and observational records of *Kolekanos* spp., including information on species, catalogue numbers, field numbers, localities, geographical coordinates and source of records. Abbreviations: California Academy of Science (CAS), Florida Museum of Natural History (UF), Kissama Foundation (FKH), National Museum of Namibia, Windhoek (NMNW), Ditsong National Museum of Natural History (formerly the Transvaal Museum; TM) and Port Elizabeth Museum (PEM). Where material was not collected, references are stated as Not Available (NA).

Species	Catalog Number	Field Number	Locality	GPS Coordinates	Source
*Kolekanosplumicaudus*	TM 40521–31	–	Tambor	-16.1355, 12.4297	Haacke (2008)
*Kolekanosplumicaudus*	TM 40553–55	–	Curoca River Crossing	-16.3027, 12.4165	Haacke (2008)
*Kolekanosplumicaudus*	TM 40755–61	–	11 km NE from Iona	-16.8606, 12.6106	Haacke (2008)
*Kolekanosplumicaudus*	PEM R18047; PEM R18010–5; CAS 248782	–	7 km NE from Iona	-16.8583, 12.6127	Vaz Pinto et al. (2021)
*Kolekanosplumicaudus*	FKH 0235	P9.254	Camp Baptista Cunene	-17.1603, 12.0182	Vaz Pinto et al. (2021)
*Kolekanosplumicaudus*	FKH 0236	P9.255	Camp Baptista Cunene	-17.1603, 12.0182	Vaz Pinto et al. (2021)
*Kolekanosplumicaudus*	UF 187219–22; CAS 262389–91	–	Omauha	-16.1996, 12.3987	Agarwal et al. (2017)
*Kolekanosplumicaudus*	FKH-0782	JLRZC0109	Omauha	-16.1987, 12,401258	Vaz Pinto et al. (2021)
*Kolekanosplumicaudus*	FKH-0343	P9.286	Omauha	-16.1996, 12.3987	Vaz Pinto et al. (2021)
*Kolekanosplumicaudus*	FKH-0344	P9.287	Omauha	-16.1996, 12.3987	Vaz Pinto et al. (2021)
*Kolekanosplumicaudus*	FKH-0345	P9.288	Omauha	-16.1996, 12.3987	Vaz Pinto et al. (2021)
*Kolekanosplumicaudus*	FKH-0346	P9.289	Omauha	-16.1996, 12.3987	Vaz Pinto et al. (2021)
*Kolekanosplumicaudus*	NA	NA	Mutuovano	-15.9153, 12.3848	Vaz Pinto et al. (2021)
*Kolekanosplumicaudus*	NA	NA	Muende-Curoca	-16.2892, 12.3180	Vaz Pinto et al. (2021)
*Kolekanosplumicaudus*	NA	NA	Tchitchaki	-16.2877, 12.2753	Vaz Pinto et al. (2021)
*Kolekanosplumicaudus*	NA	NA	Humbi	-16.9858, 12.5415	Vaz Pinto et al. (2021)
*Kolekanosplumicaudus*	NA	NA	Congundo	-17.0396, 12.6013	Vaz Pinto et al. (2021)
*Kolekanosplumicaudus*	NA	NA	Conguiungulo	-16.8437, 12.6141	Vaz Pinto et al. (2021)
*Kolekanosplumicaudus*	FKH-0534	P1.021	Maongo-Giraul	-15.0326, 12.4146	Vaz Pinto et al. (2021)
*Kolekanosplumicaudus*	FKH-0535	P1.022	Maongo-Giraul	-15.0326, 12.4146	Vaz Pinto et al. (2021)
*Kolekanosplumicaudus*	–	P1.075	Chamaleva	-15.6863, 12.6124	Vaz Pinto et al. (2021)
*Kolekanosplumicaudus*	NMNW R11011	–	Tchamalindi	-16.9752, 12.8833	Vaz Pinto et al. (2021)
*Kolekanosplumicaudus*	NMNW R11012	–	Tchamalindi	-16.9752, 12.8833	Vaz Pinto et al. (2021)
*Kolekanosplumicaudus*	–	P1.126	Cafema	-17.1289, 12.5138	Vaz Pinto et al. (2021)
*Kolekanosplumicaudus*	–	P1.127	Cafema	-17.1306, 12.5067	Vaz Pinto et al. (2021)
*Kolekanosplumicaudus*	FKH-0574	P1.115	Tchamalinde	-16.9752, 12.8833	This work
*Kolekanosplumicaudus*	FKH-0661	P1.246	Maongo	-15.0461, 12.4310	This work
*Kolekanosplumicaudus*	FKH-0662	P1.247	Maongo	-15.0461, 12.4310	This work
*Kolekanosplumicaudus*	FKH-0663	P1.248	Maongo	-15.0461, 12.4310	This work
*Kolekanosspinicaudus* sp. nov.	FKH-0645	P1.227	Carivo	-13.1923, 13.4211	This work
*Kolekanosspinicaudus* sp. nov.	MNCN 50768	P1.228	Carivo	-13.1923, 13.4211	This work
*Kolekanosspinicaudus* sp. nov.	FKH-0647	P1.229	Carivo	-13.1923, 13.4211	This work
*Kolekanosspinicaudus* sp. nov.	FKH-0648	P1.230	Carivo	-13.1923, 13.4211	This work
*Kolekanosspinicaudus* sp. nov.	FKH-0649	P1.231	Carivo	-13.1923, 13.4211	This work
*Kolekanosspinicaudus* sp. nov.	FKH-0650	P1.232	Carivo	-13.1923, 13.4211	This work
*Kolekanosspinicaudus* sp. nov.	MNCN 50766	JLRZC0212	Ekongo	-13.2494, 13.2065	This work
*Kolekanosspinicaudus* sp. nov.	FKH-0845	JLRZC0213	Ekongo	-13.2494, 13.2065	This work
*Kolekanosspinicaudus* sp. nov.	MNCN 50767	JLRZC0214	Ekongo	-13.2494, 13.2065	This work

### ﻿Phylogenetic analysis and p-distance analysis

To determine the correct placement of the species and explore diversification within *Kolekanosplumicaudus*, Bayesian Inference (**BI**) and Maximum Likelihood (**ML**) analyses were performed using the ND2 sequence alignment. The best partition scheme and best-fitting models of molecular evolution were selected using PartitionFinder v.1.1.1 (Lanfear et al. 2012). The best-fitting model scheme selected was as follows: TVM+I+G, TVM+G and TrN+I+G, by codon position. Bayesian Inference (BI) (MrBayes v.3.2.7a; Ronquist et al. 2012) was implemented on the CIPRES Science Gateway XSEDE online resource (http://www.phylo.org; Miller et al. 2010; Tamura et al. 2013). Maximum Likelihood (ML) analysis was conducted using IQ-TREE v.2.1.2 (Nguyen et al. 2015), using a random starting tree and the ultrafast bootstrap approximation (UFBoot) method (Hoang et al. 2018) with 1000 bootstrap replicates, using the gene-partitioned scheme mentioned above.

Finally, uncorrected pairwise sequence divergences (p-distance) were calculated for the ND2 sequences, in MEGA v.10.1.7 (Kumar et al. 2018) to explore intra- and interspecific variation. Standard errors (s.e.) were also calculated in MEGA v.10.1.7.

### ﻿Morphology

For this study, we examined 19 adult specimens of *Kolekanos*, collected during different expeditions and deposited in the National Museum of Natural Science (**MNCN**), Spain, Fundação Kissama (**FKH**), Angola and Port Elizabeth Museum (**PEM**), South Africa. Additionally, we reviewed the descriptions of external morphologies of *K.plumicaudus* (Haacke 2008) and other circum-Indian Ocean leaf-toed geckos (Heinicke et al. 2014), as well as osteological features provided by Heinicke et al. (2014) for all representative species within this group. The morphometric and meristic details collected were as follows: snout-vent length (**SVL**, from tip of snout to anteriorly cloacal opening), tail length (TL, from posteriorly cloacal opening to tip of tail), trunk length (**TRL**, from posterior insertion of the forelimb to anterior insertion of the hindlimb), head length (**HL**, from snout to the posterior section of the ear aperture), head width (**HW**, measured at the widest portion of the head), head height (**HH**, measured at the highest portion of the head), maximum horizontal orbital diameter (**OD**), maximum ear diameter (EarL), crus length (**CL**, from base of heel to knee), forearm length (**FL**, from insertion to the palm), nares to eye distance (**NE**, distance between anteriormost point of eye and nostril), snout to eye distance (SE, distance between anteriormost point of eye and tip of snout), eye to ear distance (EE, distance from anterior edge of ear opening to posterior corner of eye), internarial distance (IN, shortest distance between nares), interorbital distance (**IO**, shortest distance between left and right supraciliary scale rows). All measurements were taken in millimetres (mm) with a digital caliper (accuracy of 0.01 mm). The meristic data collected were: number of supralabials, number of infralabials, subdigital lamellae from the base of the digits to the leaf-toed lamellae on the first and fourth finger and toe, respectively, number of scales from the anterior part of ear opening to the posterior part of the eye, number of scales from anterior eye to nostril, number of scales between the eyes and tail ornamentation. Meristic data were collected with the help of a Leica LD2500 or Nikon SMZ1270 dissecting microscope. In order to undertake a preliminary examination of the overall morphometric variation, we performed a Principal Component Analysis (PCA), using only continuous variables, which we first log transformed and then corrected for size (by dividing the transformed data by the SVL) before the PCA analysis. We also tested the existence of variation between the two-representative taxa and between sexes of each separate taxon, using permutational ANOVAs (in the case of SVL) and ANCOVAs (in the case of the other variables, using SVL as a covariate). Both statistical analyses were performed in R v.3.6.2.

For osteological comparisons, we performed High Resolution X-ray Computed Tomography (HRCT) scans of one adult female (MNCN 50770) from the southern range and two adult males (MNCN 50769 and MNCN 50766) from the northern range of *Kolekanos*, at Centro de Instrumentación Científica of Granada (CIC), Spain, using a Zeiss Xradia 510 Versa, under the following settings: voltage = 80 kV, current = 60 μA, exposure time = 3 sec and computed voxel size (volumetric pixel) of 12.67 μm. Additionally, we examined in detail the HRCT of an adult male of *K.plumicaudus* (CAS 248782; ark:/87602/m4/M101108) provided by Heinicke et al. (2014). HRCT scans have been deposited in Morphosource (Project ID 000433817; MNCN 50766; MNCN 50769; MNCN 50770). 3D segmentation models were generated for the articulated skulls in Avizo Lite 2020.2 (Thermo Fisher Scientific 2020). To facilitate visualisation, individual bone units for skulls and jaws were coloured following the same colour pallet as Lobón-Rovira and Bauer (2021). Annotations were made in Adobe Illustrator CC 22.0.1 (Adobe Systems Incorporated 2017) following the anatomical terminology of Daza et al. (2008), Evans (2008) and Heinicke et al. (2014).

## ﻿Results

**Figure 1.**
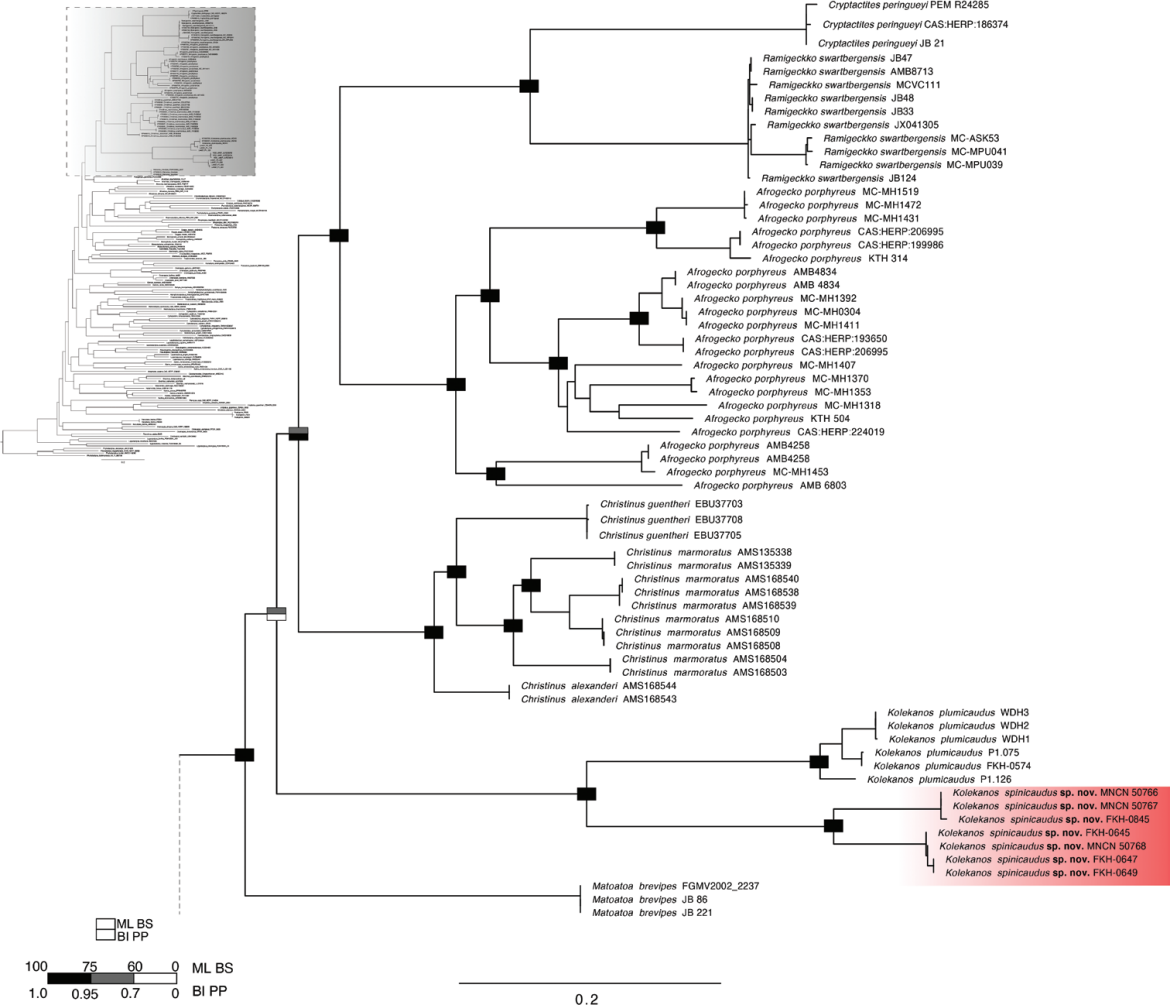
Maximum Likelihood phylogeny, with Bayesian Inference support overlaid. Support values (ML BS = Maximum Likelihood bootstrap values; BI PP = Bayesian Inference posterior probabilities) are shown graphically at the nodes according to the colours shown in the inset key. *Kolekanosspinicaudus* sp. nov. is highlighted in red.

While the two phylogenetic analyses (BI and ML) did not retrieve the same topology with regard to the deeper-level topological structuring, both were largely concordant in recovering the monophyletic circum-Indian Ocean group and recognising two clearly distinct sister taxa within *Kolekanos* (Fig. 1). The two lineages here recognised are molecularly well-differentiated, with 24.1% ND2 uncorrected p-distance from each other and regarded as separate species (Table 2). Both species presented a large disparity in the intraspecific variation. While *K.plumicaudus* showed lower intraspecific variation (3.8% ± 0.4 s.e.), with the maximum p-distance (5.2%) found between two isolated highlands in Iona National Park (Tchamalindi and Cafema), the new undescribed species presented high intraspecific diversity (7.3% ± 0.6 s.e.) between the two populations found in relatively close proximity in Benguela Province (Table 2).

**Table 2.** ND2 divergences (uncorrected pairwise distances) between circum-Indian leaf-toed geckos. Bold values depict intraspecific divergences.

ID	1	2	3	4	5	6	7	8	9	10
**1.** *Afrogeckoporphyreus*	**16.28**									
**2.** *Ramigekkoswartbergensis*	28.11	**2.55**								
**3.** *Kolekanosplumicaudus*	30.83	32.48	**3.79**							
**4.***Kolekanosspinicaudus* sp. nov.	31.33	31.43	24.49	**7.26**						
**5.** *Cryptactitesperingueyi*	28.27	23.06	32.83	31.51	**0.58**					
**6.** *Matoatoabreviceps*	26.50	28.07	29.95	30.01	28.75	**0**				
**7.** *Christinusalexanderi*	24.50	26.32	29.92	31.75	26.42	23.42	**0**			
**8.** *Christinusmarmoratus*	25.32	27.08	29.40	30.80	27.20	25.31	13.52	**7.95**		
**9.** *Christinusguentheri*	24.29	26.47	29.06	29.13	26.84	23.49	13.52	15.03	**0.32**	
**10.** *Goggialineata*	31.11	32.42	34.74	35.29	32.15	30.70	28.80	30.19	29.56	**n/c**

**Figure 2.**
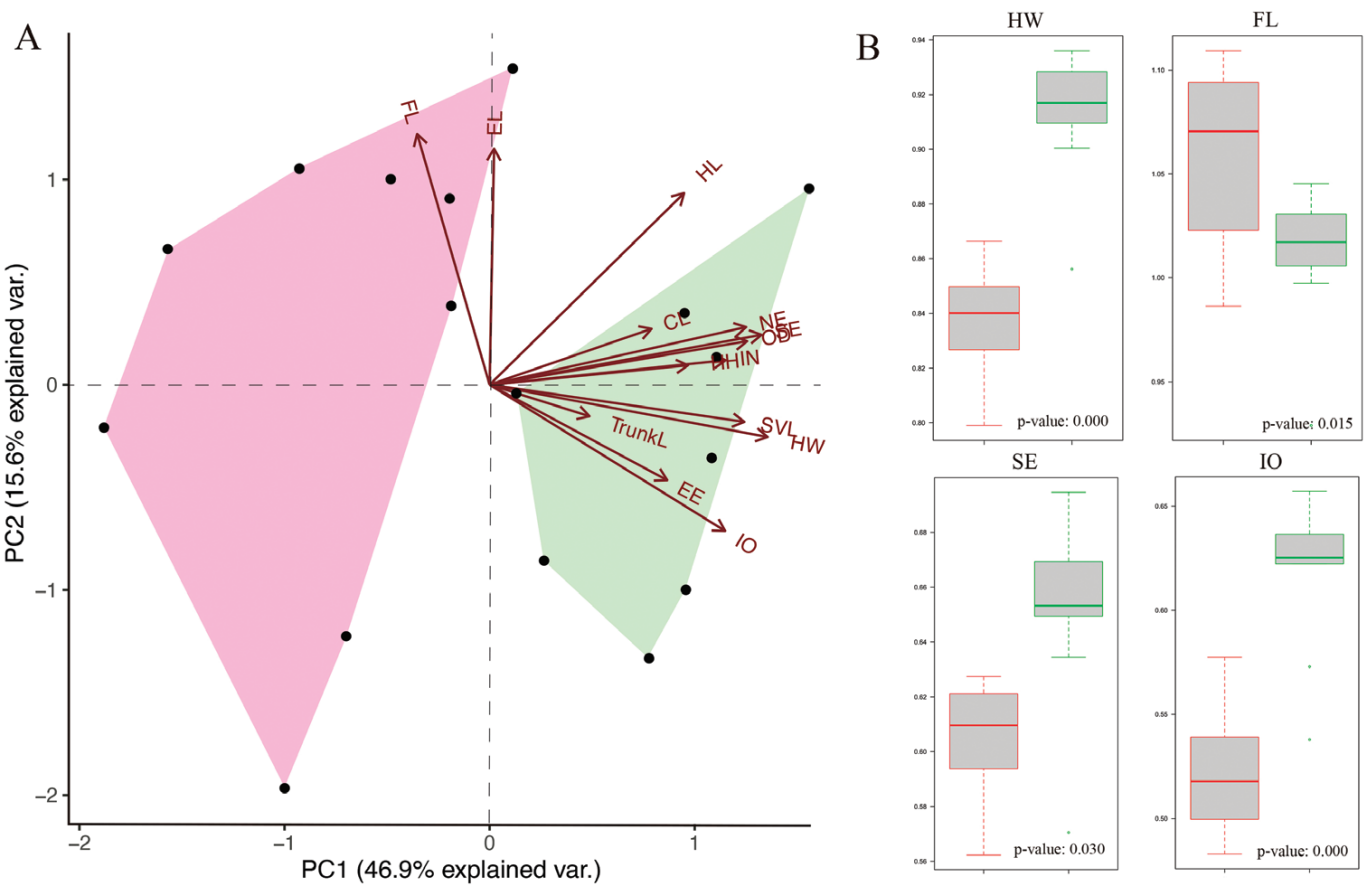
**A**PCA plots of the first principal component (PC 1) versus the second (PC 2) of morphometric analysis for the two species of *Kolekanos*. The green polygon denotes the distribution within PCAs of *K.spinicaudus* sp. nov. and the pink polygon of *K.plumicaudus*. For loadings of all axis and explained variance, see Suppl. material 3. **B** boxplots (top whisker – maximum value; lower whisker – minimum value; bold horizontal line – median; box – 1^st^ and 3^rd^ quartile) of morphological features where ANOVA t-values where ≤ 0.05; p-value of the one-way ANOVA test is indicated at the bottom of each boxplot. For abbreviations, see Material and methods section.

Morphological analysis revealed morphological differences between the two main clades within *Kolekanos*. PCA analysis explained a considerable part of the variation within these clades, with PC1 (46.9% of variation) and PC2 (15.6% of variation) showing two well-separated groups (Fig. 2A). These results are supported by the univariate morphometric analysis (ANOVA), which detected significant differences between clades (Fig. 2B), in head width (HW, F_1,14_ = 59.451, p = 0.000), forearm length (FL, F_1,14_ = 7.764, p = 0.015), snout to eye distance (SE, F_1,14_ = 5.905, p = 0.030) and interorbital distance (IO, F_1,14_ = 25.834, p = 0.000) (Suppl. material 2). Additionally, visual comparison suggested that both species could be separated also based on the robustness stage, relative to body and hindlimbs. However, we failed to retrieve any statistically significant differences from the morphometric traits analysed to confirm this visual difference (Suppl. material 2).

**Figure 3.**
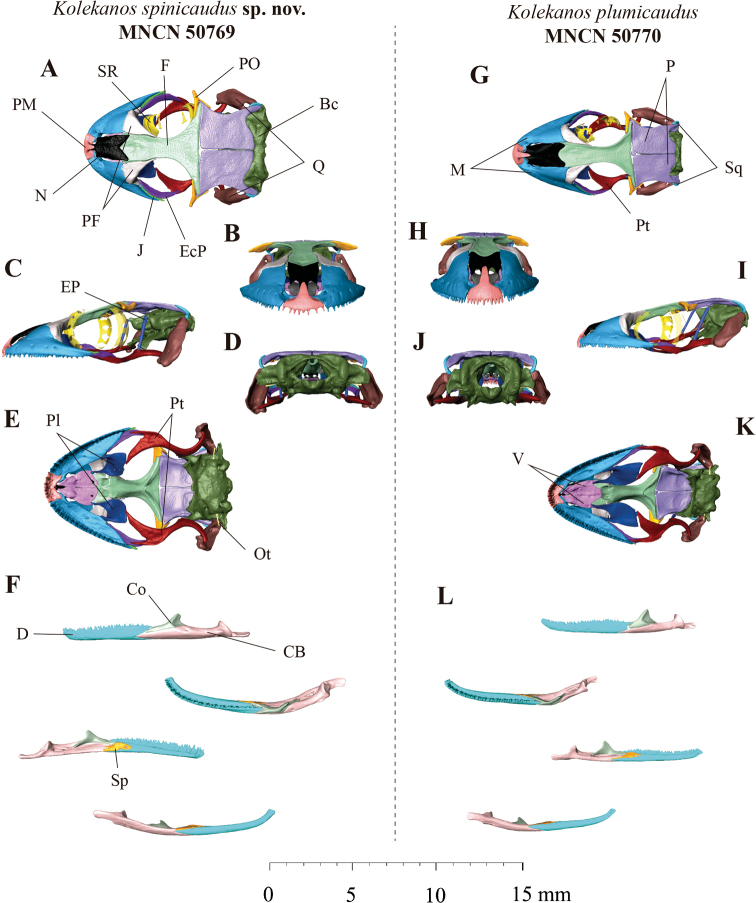
Detailed views in **A** dorsal **B** frontal **C** lateral **D** posterior and **E** ventral of skull and **F** lateral, dorsal, medial and ventral of left jaw (from top to bottom) of *K.spinicaudus* sp. nov. (MNCN50769). Detailed views in **G** dorsal **H** frontal **I** lateral **G** posterior and **K** ventral view of skull and **L** lateral, dorsal, medial and ventral of left jaw (from top to bottom) of *Kolekanosplumicaudus* (MNCN50770). Abbreviations: Bc, braincase; Co, coronoid; CB, compound bone; D, dentary; EcP, ectopterygoid; EP, epipterygoid; F, frontal; J, jugal; M, maxilla; N, nasal; Ot, otostapes; P, parietal; PF, prefrontal; Pl, palatine; PM, premaxilla; PO, postorbitofrontal; Pt, pterygoid; Q, quadrate; Sp, splenial; SR, sclerotic ring; V, vomer.

Although the osteological reconstruction demonstrated the skulls of *Kolekanos* to be very conserved, we did find differences, mostly in overall shape of the head, supporting the above morphological findings (Fig. 3). While *K.plumicaudus* presented a more slender and longer-snouted head shape (Fig. 3G), the here recognised new taxon displayed a more rounded and laterally broader head shape (Fig. 3A), with a more compressed head shape in its dorsoventral profile. This modification in the head shape seems to be reflected in osteological features in the northern clade, such as larger jugal bone, more elongated lateral process of the postorbitofrontal, more compressed premaxilla and maxilla bones in its dorsoventral view and wider in the lateral profile of the bones. It is noteworthy that the specimen CAS 248782 had a pair of nasal bones, in contrast with the three specimens analysed in this study, which presented one fused nasal bone.

Therefore, the above morphological and phylogenetic differences support the recognition of two different species within *Kolekanos* and we take the opportunity to describe the second lineage recovered as a new species below. In this manuscript, we have applied the general lineage-based species concept, where we treat all independent evolving lineages represented and supported by multiple lines of evidence, as listed above, as separate species (de Queiroz 1998).

### ﻿ Kolekanos spinicaudus sp. nov.

Taxon classificationAnimaliaSquamataGekkonidae

8448786F-208D-5B13-8D0C-0794BF71DCDE

https://zoobank.org/80186811-4C1C-4F26-B791-0824BA79E221

**Figure 4.**
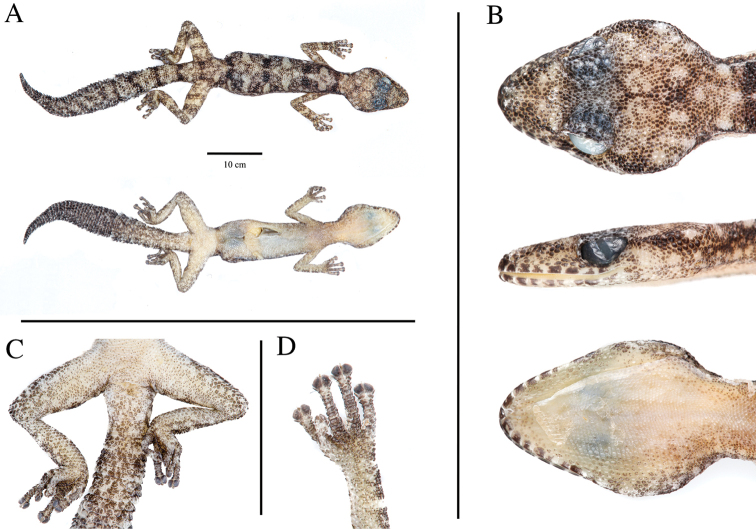
Holotype of *Kolekanosspinicaudus* sp. nov. (MNCN50769) from Carivo, Benguela Province, Angola **A** dorsal and ventral view of whole specimen **B** detail of head (from top to bottom) in dorsal, lateral and ventral views **C** detail of pelvic region and hind-limbs in ventral view **D** detail of left fingers. Photos by Alberto Sanchez Vialas (MNCN).

**Figure 5.**
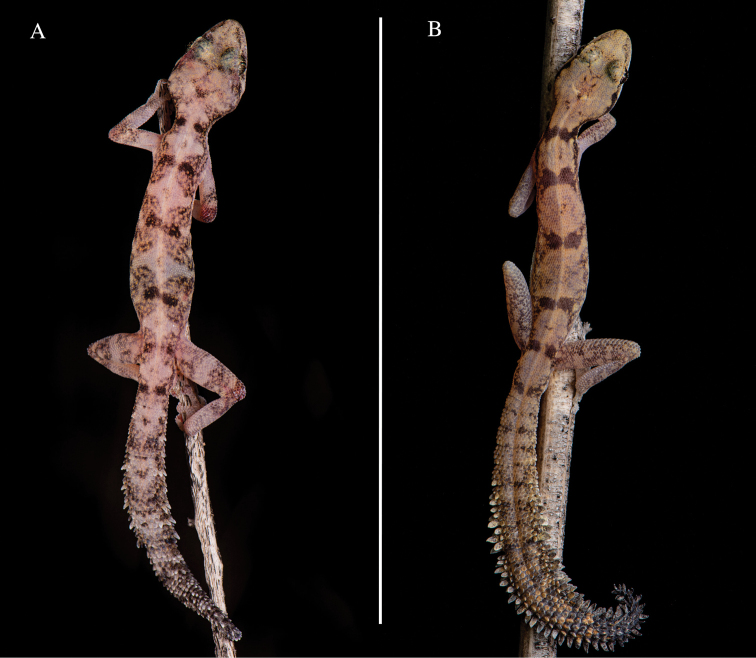
**A** dorsal view in life of *K.spinicaudus* sp. nov. from Carivo and **B***K.plumicaudus* from Omahua. Photos Javier Lobo´n-Rovira.

**Figure 6.**
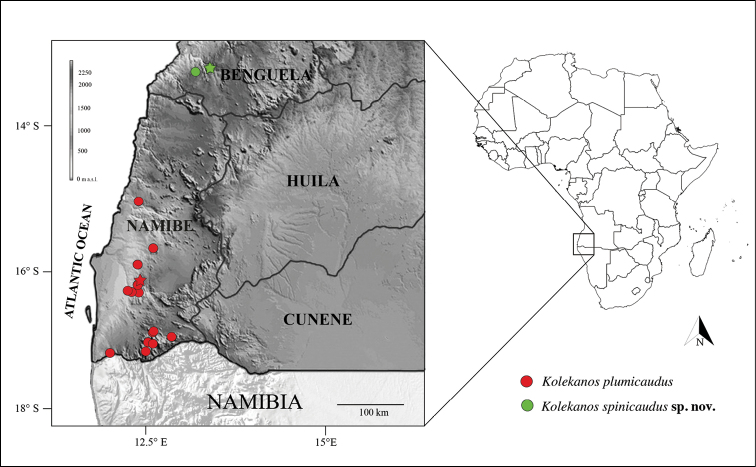
Geographical records of *Kolekanos* within Angolan territory. Red circles depict records of *K.plumicaudus*; green circles represent *K.spinicaudus* sp. nov. Stars represent type localities. Background grey scale represent elevation (Huntley and Ferrand 2019).

**Figure 7.**
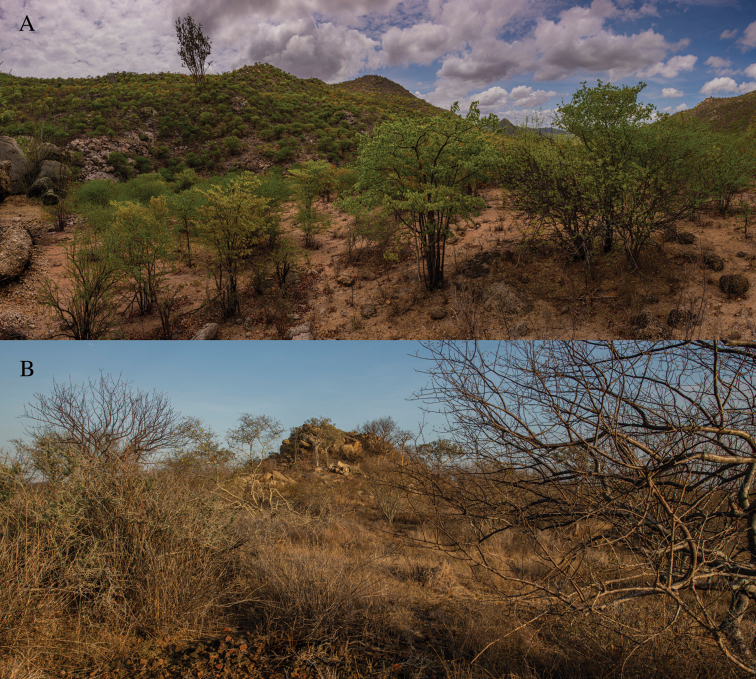
Habitat of *Kolekanosspinicaudus* sp. nov. at **A** Carivo and **B** Ekongo. Photos Javier Lobón-Rovira.

Figs 3 , 4 , 5 , 6 , 7 , Tables 3 , 4 , Suppl. material 2

#### Holotype.

MNCN 50769, adult male, with regenerated tail and incision in the ventral region, collected in Carivo (-13.19225, 13.42108, 362 m a.s.l.), Benguela Province, Angola, by Pedro and Afonso Vaz Pinto on 19 August 2021.

#### Paratypes.

MNCN 50766, adult male, collected from Ekongo (-13.24940, 13.20650, 636 m a.s.l.), Benguela Province, Angola, by Javier Lobón-Rovira and Pedro Vaz Pinto on 22 November 2021; MNCN 50767 & FKH-0845, adult females, with the same collecting data as the previous. FKH-0645 & FKH-0650, adult females, FKH-0647–8, adult males, MNCN 50768, subadult male, all with the same collecting data as the holotype.

#### Etymology.

The name “*spinicaudus*” is derived from the combination of the Latin words “spina” and “cauda”, that refers to the spiny appearance of the tail of the new species. The species epithet is used as a singular nominative adjective “-*us*”.

#### Diagnosis.

*Kolekanos* can be easily differentiated from other circum-Indian leaf-toed and African leaf-toed geckos, based on its ornamented tail (versus non-ornamented tail in the remaining genera). The new species differs from *K.plumicaudus*, based on the following characters: different ornamentation of the tail, being composed by modified scales on the margins of the original tail which resemble white lateral spines (versus feathered-like tail in *K.plumicaudus*); broader head (minimum HW = 7.95 mm versus maximum HW = 7.35 mm in *K.plumicaudus*); more robust body, with shorter forelimbs (versus thinner and more slender body in *K.plumicaudus*, Fig. 5); proportionally larger snout to eye distance (SE mean 4.48 mm ± 0.34 s.e. versus 3.99 mm ± 0.22 s.e. in *K.plumicaudus*) and interorbital distance (IO mean 4.14 mm ± 0.34 s.e. versus 3.33 mm ± 0.28 s.e. in *K.plumicaudus*); and dorsal pattern is less contrasted, based on zig-zag black patches surrounded by lighter patches (versus dark blocks well contrasted, not surrounded by lighter patches in *K.plumicaudus*). The new species can also be differentiated from *K.plumicaudus* by the following osteological characteristics: 1) larger jugal bone (versus reduced jugal); 2) more prominent lateral process of the postorbitofrontal (versus less prominent lateral process of postorbitofrontal); 3) more compressed premaxilla and maxilla bone on its dorsoventral profile and wider in the lateral profile of the bones; 4) ascending process of the premaxilla shorter (versus more elongated); 5) braincase compressed dorsoventrally (versus more rounded in *K.plumicaudus*); 6) palatine length and width equal (versus unequal); 7) postero-lateral process of parietal rounded and slightly curved (versus flat postero-lateral process of parietal broad and flat that curves downwards posteriorly); 8) anterolateral process of the coronoid markedly enlarged (versus more reduced anterolateral process). *Kolekanosspinicaudus* sp. nov. also differs from *K.plumicaudus* by circa 24% (uncorrected p-distance) ND2 mitochondrial DNA.

#### Holotype description.

**(Fig. 4).** Measurements and meristic characters of the holotype are presented in Table 3. Adult male with a SVL of 44.59 mm and partially (2/3) regenerated tail, tail length (TL) 36.77 mm. Body moderately slender, nape distinct. Head slightly broader than the body and markedly compressed dorsoventrally (HH/HL = 0.27). Canthus rostralis smooth, almost absent. Eye diameter (2.35 mm), with vertical pupil and crenulated margin. Supraciliar scales small and rounded. Ear height (0.47 mm). Ear to eye distance larger than eye diameter (3.72 mm). Snout rounded and slightly pointed. Body relatively slender and elongated (TrunkL/SVL = 0.44). Fore- and hindlimbs moderate and stout, forearm large (FL/SVL 0.23), tibia short (CL/SVL 0.18). Digits elongated and clawed. All digits of manus and pes indistinctly webbed. All digits with granular basal scales and more distal widened divided lamellae. One pair of leaf-like terminal scansors. Number of scansors: 7-10-10-11-10 (right manus) and 7-10-10-11-10 (left manus)/7-9-11-11–10 (right pes) and 7-9-11-11–9 (left pes). Relative length of digits manus I < II < III < IV > V and pes I < II < III > IV > V. Scalation: Frontal scales granular and larger than occipital scales. Occipital scales small and granular. Rostral in direct contact with nostrils, 1^st^ supralabials, supranasals and one internasal scales. 8/8 supralabial and 9/9 infralabials. First supralabial in contact with the nostril. Nostril circular and surrounded by rostral, 1^st^ supralabial, supranasal and three reduced postnasals. Lower postnasal half the size of the upper postnasal and supranasal. Two rows of scales between supralabials and the orbit. Mental triangular and rounded posteriorly, with two small rounded postmental scales. 1^st^ infralabial rectangular and slightly larger than mental. Gular scales small and granular. Ventral scales small and granular. Precloacal pores absent. The dorsal pholidosis present homogenous granular scales from head to tail. The first third of the original tail presents lateral whitish “spine-like” scales, being absent in the last portion of the tail. Post-cloacal scales slightly larger and quadrangular. Osteology: the skull (Fig. 3) displays no co-ossification with the overlying skin. Nasals are fused. Single frontal. Paired parietals. Stapes imperforate. 14 scleral ossicles. 11 premaxillary tooth loci. 36–38 maxillary and 38 dentary tooth loci. Braincase elements fused. Postorbitofrontal arrow-shaped, with lateral process as long as anterior and posterior process. Parietal wider than longer. Jugal small, but visible.

**Table 3.** Morphological (morphometric and meristic) of *Kolekanosspinicaudus* sp. nov. Measurements are represented in millimetres (mm). For abbreviations, see Material and methods section. R = regenerated tail, M = male, F = female.

Species	*K.spinicaudus* sp. nov.	*K.spinicaudus* sp. nov.	*K.spinicaudus* sp. nov.	*K.spinicaudus* sp. nov.	*K.spinicaudus* sp. nov.	*K.spinicaudus* sp. nov.	*K.spinicaudus* sp. nov.	*K.spinicaudus* sp. nov.	*K.spinicaudus* sp. nov.
Catalogue#	MNCN 50769	FKH0845	MNCN 50767	MNCN 50766	FKH-0645	FKH-0648	FKH-0647	FKH-0650	MNCN 50768
Status	Holotype	Paratype	Paratype	Paratype	Paratype	Paratype	Paratype	Paratype	Paratype
Sex	M	F	F	M	F	M	M	F	M
SVL	44.59	43.19	44.15	40.82	43.71	41.66	42.72	41.65	35.87
TAL	R 36.77	–	R 22.26	R 39.76	R 31.39	36.85	R 28.85	–	39.44
TrunkL (mm)	19.82	17.87	18.65	16.85	19.36	18.44	19.80	18.27	16.71
HL (mm)	10.99	11.67	11.6	11.09	11.10	10.76	11.06	10.88	9.59
HW (mm)	8.63	8.12	8.56	8.48	8.28	7.95	8.21	8.26	7.18
HH (mm)	2.95	4.20	3.80	4.00	3.52	3.77	3.86	3.60	3.17
OD	2.35	2.35	2.50	2.51	2.62	2.35	2.62	2.21	2.08
EL	0.47	0.65	0.85	0.69	0.76	0.64	0.48	0.47	0.54
CL	8.03	8.59	9.01	8.74	8.15	8.01	7.80	8.15	7.94
FL	10.39	10,.68	11.10	9.94	10.93	10.73	10.13	10.40	8.49
NE	3.30	3.40	3.75	3.44	3.61	3.35	3.55	3.43	2.99
SE	4.48	4.50	4.95	4.68	4.56	4.31	4.67	4.46	3.72
EE	3.72	3.21	3.65	3.76	3.66	3.31	3.57	3.10	3.12
IN	1.55	1.55	1.48	1.45	1.45	1.40	1.34	1.39	1.22
IO	4.29	4.33	4.22	4.54	4.33	3.45	4.19	4.21	3.74
N° lamellae 1^st^ toe (Right/Left)	4/3	5/5	4/5	4/5	4/4	4/4	4/3	4/3	5/4
N° lamellae 4^th^ toe (Right/Left)	5/5	7/5	6/6	6/5	6/7	5/5	6/5	6/6	6/6
N° lamellae 1^st^ finger (Right/Left)	3/4	4/	4/4	3/3	4/4	5/4	4/3	3/4	3/3
N° lamellae 4^th^ finger (Right/Left)	6/7	5/6	6/7	6/6	5/6	5/6	6/4	5/7	7/6
N° postmental	2	2	2	2	2	2	2	2	2
N° infralabial	9	8	8	9	9	9	9	9	9
N° supralabial	8	10	10	10	9	9	10	9	9
N° internasal	1	1	1	1	1	1	1	2	1
N° scales ear to eye	17	18	16	17	12	13	14	15	16
N° scales eye to nostril	11	13	11	12	11	10	11	10	12
N° scales eye to eye	17	20	15	16	16	15	17	18	16

#### Variation.

Variation in scalation and body measurements of the paratypes of *K.spinicaudus* sp. nov. are reported in Table 3. All the material analysed agrees with the holotype description with the exception of the tooth loci, where the specimen MNCN 50766 presented a larger number in the tooth loci of maxilla and dentary (> 40).

#### Colouration.

***In life*** (Fig. 5A): dorsal colouration varies from light pinkish to light brown, with black spots surrounded by lighter brownish regions disposed in zig-zag, from nape to tail. Dorsal reticulated light brownish colouration on tail and fore- and hind-limbs. Anterior part of the tail with marked hourglass-shaped pattern. Ventrum uniformly light cream pink from snout to posterior region of the cloaca. Tail slightly darker than the dorsum dorsally, being even darker in the ventral section, with white lateral spine-like scales on original tail. Last fourth portion of tail black. ***In preservative*** (After 4 months in preservation; Fig. 4): dorsal pattern persistent as “*in life*” with dorsal colouration whitish-greyish. Dark section more marked.

#### Distribution.

**(Fig. 6)**. This species has only been found at two sites in a very restricted region, in southern Benguela Province. The area lies above the first elevational range recognised for southern Angola’s orographic relief, with specimens retrieved between 400 m and 650 m a.s.l. It can be broadly characterised as a rugged and transitional semi-arid landscape, albeit more vegetated and less arid than the coastal lowlands to the west and less mountainous and forested than eastern regions neighbouring the great escarpment. Despite its unique and unmistakable features, this species had eluded previous surveys conducted in coastal Benguela Province. In the last 5 years our team visited the same area at least five times preceding the discovery, spending at least two days per survey. Even though we found the species to be relatively common at the two referred sites, we failed to confirm its presence in several other locations with presumably suitable habitat, suggesting that it might be highly specialised and sensitive to local environmental conditions. It is possible for the species to be more common and widely distributed in poorly-surveyed regions to the southeast or north of its known range and we recommend further surveys in the region to address the conservation status of this poorly-known species.

#### Habitat and natural history notes.

**(Fig. 7)**. The local habitat, at both sites where the species was discovered, seems to be a transitional zone in coastal Angola, displaying a rich vegetation mosaic of acacia and mopane savannah, including *Senegaliamellifera*, *Senegalia* spp., *Colophospermummopane*, *Terminalia prunioides*, *Commiphora* spp. and presence of succulents, such as *Euphorbia* spp. and *Aloelittoralis*. In contrast, and despite being known from a relatively wider region and across considerable elevational ranges, *K.plumicaudus* is found in much more arid and sparsely vegetated environments. The new species was mostly found at night foraging in the ecotone between the trees/bushes and moderate to large granite boulders. One individual (not collected) was retrieved while sheltering under a rock flake during the day, behaviour which has been documented for the closely-related *K.plumicaudus* (Agarwal et al. 2017; Vaz Pinto et al. 2021). When not stretched horizontally, this species curls the tail laterally, but not upwards, while *K.plumicaudus* often erects the tail upwards and may wave the tip (Agarwal et al. 2017). The first individual observed was seen running fast on the ground between a granite boulder and a tree, but more often, they were found perched on branches and once on a grass stem. Unlike *K.plumicaudus*, which readily jumps amongst thin branches when disturbed, *K.spinicaudus* sp. nov. seems to prefer to run along thicker branches or drop to the ground and run for safety. Two individuals were observed mating at night (18 August 2021 19 h 55 m) on a thin branch of *Salvadorapersica*. One female specimen (FKH-0645) collected in November 2021 contained two well-developed eggs. This species has been found in syntopy with another Angolan leaf-toed gecko, *Baueriusansorgii*. Finally, due to the complex biogeography of Angola, an updated and stabilized biogeographic classification, especially for south-western Angola, is still lacking. Current schemes depend on the authors interpretation and underlying data used (e.g. phytocoria, centres of endemism, realms, biomes, ecoregions) resulting in different units recognised and sharp boundaries (Burgess et al. 2004; Dinerstein et al. 2017) which often do not match the situation on the ground. Thus, we cannot currently assign any specific biogeographic region to any of these two taxa and are anticipating a better review of Angolan biogeographic units through Huntley (in prep.) in the near future.

**Table 4.** Morphological (morphometric and meristic) of *Kolekanosplumicaudus*. Measurements are represented in millimetres (mm). For abbreviations, see Material and methods section, R = regenerated, M = male, F = female.

Species	*K.plumicaudus*	*K.plumicaudus*	*K.plumicaudus*	*K.plumicaudus*	*K.plumicaudus*	*K.plumicaudus*	*K.plumicaudus*	*K.plumicaudus*	*K.plumicaudus*	*K.plumicaudus*
Catalogue#	MNCN 50770	FKH-0661	FKH-0663	PEM R18010	PEM R18014	PEM R18011	PEM R18015	PEM R18012	PEM R18013	PEM R18047
Status	–	–	–	–	–	–	–	–	–	–
Sex	F	F	F	F	M	M	F	F	F	M
SVL	41.91	38.45	40.22	42.05	42	36.57	41.84	36.97	41.84	37.38
TAL	R 34.14	R 34.38	41.91	40.34	–	R 26.35	–	33.34	–	34.68
TrunkL (mm)	19.08	17.84	19.07	18.35	18.38	16.20	18.41	16.97	19.38	20.03
HL (mm)	10.04	9.42	9.89	11.11	11.17	10.59	11.59	10.22	11.53	10.98
HW (mm)	7.03	6.91	6.81	6.71	7.07	6.29	7.13	6.92	7.35	6.70
HH (mm)	3.50	3.38	3.27	3.45	3.19	2.76	3.62	2.90	3.21	30.70
OD	2.08	2.28	2.17	2.35	2.31	2.11	2.34	2.14	2.42	2.10
EL	0.59	0.49	0.50	0.71	0.79	0.58	0.63	0.87	0.75	0.62
CL	8.15	7.39	8.11	8.90	8.34	7.33	7.45	8.05	8.41	7.85
FL	10.75	8.77	9.69	10.38	11.76	10.54	12.42	11.85	12.86	12.66
NE	2.91	2.80	2.97	3.15	3.23	3.15	3.49	2.83	3.32	3.06
SE	3.89	3.61	3.65	4.18	4.07	3.98	4.13	3.92	4.24	4.18
EE	3.71	3.00	3.49	3.47	3.45	2.91	3.49	3.19	3.25	2.96
IN	1.30	1.29	1.24	1.30	1.21	1.11	1.25	1.26	1.45	1.43
IO	3.71	3.57	3.78	3.16	3.06	3.03	3.29	3.19	3.46	3.04
N° lamellae 1^st^ toe (Right/Left)	4/4	3/3	4/3	4/4	4/4	4/4	4/4	6/6	5/4	4/4
N° lamellae 4^th^ toe (Right/Left)	6/5	5/6	5/6	6/6	7/7	6/6	7/4	5/6	6/7	7/8
N° lamellae 1^st^ finger (Right/Left)	3/4	3/4	3/4	4/4	4/4	4/4	4/4	4/4	4/4	4/4
N° lamellae 4^th^ finger (Right/Left)	d/6	6/6	6/6	6/6	8/8	7/7	8/8	7/7	7/7	7/7
N° postmental	2	1	2	3	2	2	2	3	2	2
N° infralabial	8	8	9	9	9	9	9	9	9	9
N° supralabial	10	10	10	10	9	10	9	10	9	10
N° internasal	1	1	1	0	1	0	0	1	1	1
N° scales ear to eye	13	14	15	17	17	17	18	16	15	17
N° scales eye to nostril	11	9	11	10	9	10	8	8	8	9
N° scales eye to eye	15	14	15	16	18	15	14	15	15	14

#### Conservation status.

The species seems relatively common, but highly localised. Although the general habitat does not appear to be threatened, more research is needed to confirm if the species’ distribution is larger than currently known. Therefore, following the IUCN Red List guidelines (IUCN 2022), the species should be considered as Data Deficient (DD).

## ﻿Discussion

Using molecular and morphological evidence, we herein described a new leaf-toed gecko, from southern Benguela Province, Angola, *Kolekanosspinicaudus* sp. nov., thereby adding another species to the growing list of gekkonids described in the last decade from this poorly-known African country (Ceríaco et al. 2020a, b; Marques et al. 2020; Branch et al. 2021; Lobón-Rovira et al. 2021; Conradie et al. 2022b). The recognition of *K.spinicaudus* sp. nov. as a sister species of *K.plumicaudus*, contradicts the previous knowledge of mainland circum-Indian Ocean leaf-toed geckos as monotypic genera (Lobón-Rovira et al. 2022a) and reinforces the need of further investigation on the potential cryptic diversification within another related species, such as *Afrogeckoporphyreus* (Heinicke et al. 2014). Therefore, we provide a new perspective for future work within this group, which may improve the knowledge regarding Angolan and western African gekkonid diversity.

The molecular analysis, provided in this work, has shown a large divergence of the ND2 mitochondrial gene between *K.spinicaudus* sp. nov. and *K.plumicaudus*, being even higher than the molecular divergence found between closely-related genera, such as with *Rammigekko* and *Cryptactites*. However, the external morphological similarities between these two taxa support differentiation only at species level. This high divergence can be explained by the ancient character of this group (Heinicke et al. 2014), having persisted through extreme climatological and environmental changes and, consequently, experiencing long isolation periods in southwest Africa (Chase et al. 2009; Garzanti et al. 2018). Both species revealed notable genetic intraspecific variation between close localities, which can, in both cases, be explained by the high ecological specialisation in these geckos, promoting and maintaining isolation and by the relatively fast mtDNA evolutionary rates (Jesus et al. 2006).

Regarding interspecific variation, both species have exhibited a high degree of morphological and ecological differentiation. While *K.plumicaudus* presented a more slender head and body and seems more strongly associated with the more arid environments of the Angolan Kaokoveld Desert in Namibe Province, the sister species, *K.spinicaudus* sp. nov. presented a more robust head, body and limbs and is apparently only found in the semi-arid savannahs of Benguela Province (Lobón-Rovira et al 2021). The two species are quite agile and often found foraging in bushes. *Kolekanosplumicaudus* sometimes wag their tail semi-erected when disturbed and readily jumps amongst thin branches as a primary escape strategy (Agarwal et al. 2017; Vaz Pinto et al. 2021), while *K.spinicaudus* sp. nov., when threatened, is primarily a swift runner either along branches or on the ground. These behavioural differences may likely reflect subtle adaptations to local conditions, including vegetation cover, predation, foraging and sheltering habits. In addition, both species present a distinctive tail ornamentation that can be used as a clear morphological diagnostic feature between them. These findings seem to be consistent with the idea that habitat diversity leads to species and morphological diversification (Losos and Parent 2009; Tejero-Cicuéndez et al. 2021).

Some of the osteological features provided by Heinicke et al. (2014) seem to be non-homoplastic apomorphic characters for *Kolekanos* genus, such as ectopterygoid width more or less constant along the length of the bone, prootic contacting the epipterygoid far behind from the posterior process of the postorbitofrontal and groove associated with the surangular foramen and coronoid abutting the dentary. We failed to recover some of the proposed characters for either of the two species of *Kolekanos*, such as jugal bone being very reduced, almost vestigial (versus moderated size) and anterolateral corner of parietal not clasping the frontal (versus clasping anterolateral section). Furthermore, the *K.plumicaudus* CT-scanned in this work was not fully concordant with the diagnosis presented in the description of this unique genus (Heinicke et al. 2014), for example, fused nasal bones and well-developed postorbitofrontal bone (versus unfused nasal bones and reduced, almost vestigial, postorbitofrontal bone, Heinicke et al. 2014). However, this difference could be associated with sexual dimorphism, since the only two specimens of *K.plumicaudus* represent one of each sex. Thus, this work underlines the importance of using larger series of material to fully infer diagnostic characters between species (Lobón-Rovira et al. 2021) and these being even more important to infer osteological variability (Bochaton et al. 2018), including sexual dimorphism. While we consider that the external diagnostic characters are sufficient to identify these species, we suggest caution while using the osteological differences to distinguish these two taxa due to the small sampling size available.

We here provide another example of diversification in south-western Angola, leading to speciation in the more arid desert ecosystems of Namibe Province and in the semi-arid coastal savannahs of Benguela Province, a pattern that has been found in other studies (e.g. *Hemidactylusbenguellensis*-group, Lobón-Rovira et al. 2021). Our findings underline the remarkable herpetological value of coastal Benguela Province and particularly as a potential gekkonid hotspot. We have confirmed at Carivo that at least 14 species, representing all eight Angolan genera, are living in sympatry. This is the highest number of gekkonid species recorded in a single site in the country. In addition, we also report, for the first time, the two endemic Angolan leaf-toed gecko genera (*Bauerius* and *Kolekanos*) found in syntopy in both localities where *K.spinicaudus* sp. nov. has been found.

To conclude, we recommend additional surveys in Benguela Province to study the distribution and abundance of this new species to assess its conservation status and further research is needed in northern Namibe Province to explore potential contact zones between the two *Kolekanos* species. Due to the high genetic divergence between the two recorded populations of *K.spinicaudus* sp. nov., we also suggest caution when addressing conservation strategies in western Angola, since it may affect ongoing speciation processes within *Kolekanos* in this region.

## Supplementary Material

XML Treatment for Kolekanos spinicaudus

## Supplementary materials

Supplementary material 1 Additional genetic material used for this work with their corresponding field numbers, catalog numbers and GenBank accession numbers Data type Genetic material. Explanation note AMB – A. M. Bauer field series; AMS – Australian Museum; CAS – California Academy of Sciences; CHL – Colecção Herpetologica do Lubango, Angola; EBU – Evolutionary biology unit, Australian Museum; ERP – E.R. Pianka field series; FB – Francois Becker field series; FG/MV – F. Glaw/M. Vences field series; FKH – Fundação Kissama, Angola; FLMNH – Florida Museum of Natural History; JB – J. Boone personal collection; JVV – J.V. Vindum field series; GVH – G. Haagner field series; KTH – K. Tolley field series; MC – M. Cunningham personal collection; MCZ – Museum of Comparative Zoology, Harvard University; MNCN – Museo Nacional de Ciencias Naturales, Madrid, Spain; MVZ – Museum of Vertebrate Zoology, University of California, Berkeley; PEM – Port Elizabeth Museum (Bayworld); RAH – R.A. Hitchmough field series; ROM – Royal Ontario Museum; SAMA – South Australian Museum; TG – T. Gamble field series; WDH – W.D. Haacke personal collection; WRB – W.R. Branch field series; WC – W. Conradie field series; YPM – Yale Peabody Museum; ZSM – Zoologische Staatssammlung München. Dash (–) indicate no sequences available. https://binary.pensoft.net/file/764852This dataset is made available under the Open Database License (http://opendatacommons.org/licenses/odbl/1.0/). The Open Database License (ODbL) is a license agreement intended to allow users to freely share, modify, and use this Dataset while maintaining this same freedom for others, provided that the original source and author(s) are credited.Javier Lobón-Rovira, Werner Conradie, Ninda L. Baptista, Pedro Vaz Pinto

Supplementary material 2 Results of the analysis of morphometric differences between *Kolekanos* spp. and sexes Data type Morphological results. Explanation note Analyses were performed using permutational ANOVAs (for SVL, indicated as an *, and with an associated degrees of freedom of 1,14) and ANCOVAs (all the other variables, using SVL as a covariate). Significant results (p-values < 0.05) are highlighted in bold. For abbreviations see Material and methods section. https://binary.pensoft.net/file/764853This dataset is made available under the Open Database License (http://opendatacommons.org/licenses/odbl/1.0/). The Open Database License (ODbL) is a license agreement intended to allow users to freely share, modify, and use this Dataset while maintaining this same freedom for others, provided that the original source and author(s) are credited.Javier Lobón-Rovira, Werner Conradie, Ninda L. Baptista, Pedro Vaz Pinto

Supplementary material 3 Principal Component Analysis (PCA) loadings for each morphological variable measured in of *Kolekanos* spp., including standard deviation (SD), percentage of variance (% Variance) and cumulative proportion for each component. Data type Statistics. Explanation note For abbreviations, see Materials and Methods section. https://binary.pensoft.net/file/764854This dataset is made available under the Open Database License (http://opendatacommons.org/licenses/odbl/1.0/). The Open Database License (ODbL) is a license agreement intended to allow users to freely share, modify, and use this Dataset while maintaining this same freedom for others, provided that the original source and author(s) are credited.Javier Lobón-Rovira, Werner Conradie, Ninda L. Baptista, Pedro Vaz Pinto
