# Supplementary material for: ﻿A new species of feather-tailed leaf-toed gecko, Kolekanos Heinicke, Daza, Greenbaum, Jackman, Bauer, 2014 (Squamata, Gekkonidae) from the poorly explored savannah of western Angola
Source: Zookeys. 2022 Nov 2;1127:91–116. doi: 10.3897/zookeys.1127.84942 (PMC9836571; doi:10.3897/zookeys.1127.84942)
Supplement: Supplementary material 1 — Additional genetic material used for this work with their corresponding field numbers, catalog numbers and GenBank accession numbers [file zookeys-1127-091_article-84942__-s001.docx]

**Table S1.** Additional genetic material used for this work with their corresponding field numbers, catalog numbers and GenBank accession numbers. AMB – A. M. Bauer field series; AMS – Australian Museum; CAS – California Academy of Sciences; CHL – Colecção Herpetologica do Lubango, Angola; EBU – Evolutionary biology unit, Australian Museum; ERP – E.R. Pianka field series; FB – Francois Becker field series; FG/MV – F. Glaw/M. Vences field series; FKH – Fundação Kissama, Angola; FLMNH – Florida Museum of Natural History; JB – J. Boone personal collection; JVV – J.V. Vindum field series; GVH – G. Haagner field series; KTH – K. Tolley field series; MC – M. Cunningham personal collection; MCZ – Museum of Comparative Zoology, Harvard University; MNCN – Museuo Nacional de Ciencias Naturales, Madrid, Spain; MVZ – Museum of Vertebrate Zoology, University of California, Berkeley; PEM – Port Elizabeth Museum (Bayworld); RAH – R.A. Hitchmough field series; ROM – Royal Ontario Museum; SAMA – South Australian Museum; TG – T. Gamble field series; WDH – W.D. Haacke personal collection; WRB – W.R. Branch field series; WC – W. Conradie field series; YPM – Yale Peabody Museum; ZSM – Zoologische Staatssammlung München. Dash (–) indicate no sequences available. Four species of *Phyllodactylus* genus are included as outgroup, selected as representants of the family Phyllodactylidae sister to Gekkonidae family.

| **Species** | **Field/ Catalog Numbers** | **ND2** |
| --- | --- | --- |
| *Kolekanos plumicaudus* | WDH 1 | JX041304 |
| *Kolekanos plumicaudus* | WDH 2 | KF666791 |
| *Kolekanos plumicaudus* | WDH 3 | KF666792 |
| *Kolekanos plumicaudus* | P1.075 | OP605738 |
| *Kolekanos plumicaudus* | P1.126 | OP605736 |
| *Kolekanos plumicaudus* | FKH-0574 | OP605737 |
| *Kolekanos spinicaudus* **sp. nov.** | FKH-0645 | OP605742 |
| *Kolekanos spinicaudus* **sp. nov.** | MNCN 50768 | OP605741 |
| *Kolekanos spinicaudus* **sp. nov.** | FKH-0647 | OP605740 |
| *Kolekanos spinicaudus* **sp. nov.** | FKH-0649 | OP605739 |
| *Kolekanos spinicaudus* **sp. nov.** | MNCN 50766 | OP605745 |
| *Kolekanos spinicaudus* **sp. nov.** | FKH-0845 | OP605744 |
| *Kolekanos spinicaudus* **sp. nov.** | MNCN 50767 | OP605743 |
| *Afroedura africana* | MCZ R186011 | KM073619 |
| *Afroedura karroica* | PEM ZAF1112 | JX041302 |
| *Afroedura loveridgei* | GVH 3969 | JX041303 |
| *Afrogecko porphyreus* | CAS 206995 | EF490776 |
| *Afrogecko porphyreus* | AMB 4258 | KF666772 |
| *Afrogecko porphyreus* | AMB 4834 | KF666774 |
| *Afrogecko porphyreus* | MC(MH1519) | KF666790 |
| *Afrogecko porphyreus* | MC(MH1407) | KF666785 |
| *Afrogecko porphyreus* | MC(MH1392) | KF666784 |
| *Afrogecko porphyreus* | MC(MH1370) | KF666783 |
| *Afrogecko porphyreus* | MC(MH1353) | KF666782 |
| *Afrogecko porphyreus* | MC(MH1318) | KF666781 |
| *Afrogecko porphyreus* | MC(MH0304) | KF666780 |
| *Afrogecko porphyreus* | KTH 504 | KF666779 |
| *Afrogecko porphyreus* | KTH 314 | KF666778 |
| *Afrogecko porphyreus* | CAS 193650 | KF666777 |
| *Afrogecko porphyreus* | AMB 6803 | KF666776 |
| *Afrogecko porphyreus* | CAS 224019 | KF666775 |
| *Afrogecko porphyreus* | AMB 4834 | KF666774 |
| *Afrogecko porphyreus* | AMB 4258 | KF666772 |
| *Afrogecko porphyreus* | CAS 199986 | KF666771 |
| *Afrogecko porphyreus* | MC(MH1472) | KF666789 |
| *Afrogecko porphyreus* | MC(MH1453) | KF666788 |
| *Afrogecko porphyreus* | MC(MH1431) | KF666787 |
| *Afrogecko porphyreus* | MC(MH1411) | KF666786 |
| *Ailuronyx seychellensis* | PL17 | KY038014 |
| *Ailuronyx tachyscopaeus* | MCZ F38717 | JX041307 |
| *Ailuronyx trachygaster* | AMB 8160 | JX041308 |
| *Alsophylax pipiens* | CAS HERP238805 | JX041309 |
| *Bauerius ansorgii* | P8-23/ FKH 0009 | OM885010 |
| *Bauerius ansorgii* | P8-24/ FKH 0010 | OM885011 |
| *Bauerius ansorgii* | NB824/ CHL 824 | OM885012 |
| *Blaesodactylus antongilensis* | ZSM 410 | KM073686 |
| *Blaesodactylus sakalava* | WRB M18 | EU054251 |
| *Calodactylodes illingworthorum* | AMB 7415 | JX041318 |
| *Chondrodactylus bibronii* | CAS 201841 | JN543886 |
| *Chondrodactylus fitzsimonsi* | MCZ R185712 | JN393945 |
| *Christinus alexanderi* | AMS 168544 | KF666813 |
| *Christinus alexanderi* | AMS 168543 | KF666812 |
| *Christinus guentheri* | EBU 37703 | KF666800 |
| *Christinus guentheri* | EBU 37708 | KF666802 |
| *Christinus guentheri* | EBU 37705 | KF666801 |
| *Christinus marmoratus* | AMS 168540 | KF666811 |
| *Christinus marmoratus* | AMS 168539 | KF666810 |
| *Christinus marmoratus* | AMS 168538 | KF666809 |
| *Christinus marmoratus* | AMS 168510 | KF666808 |
| *Christinus marmoratus* | AMS 168509 | KF666807 |
| *Christinus marmoratus* | AMS 168508 | KF666806 |
| *Christinus marmoratus* | AMS 168504 | KF666805 |
| *Christinus marmoratus* | AMS 168503 | KF666804 |
| *Christinus marmoratus* | AMS 135338 | KF666803 |
| *Cnemaspis africana* | CAS HERP 168872 | JX041323 |
| *Cnemaspis dickersonae* | MTSN 8604 | JX041324 |
| *Cnemaspis uzungwae* | MTSN 5603 | JX041329 |
| *Cnemaspis gemunu* | AMB 7495 | KY037998 |
| *Cnemaspis podihuna* | AMB 7449 | KY038006 |
| *Cnemaspis punctata* | AA 80 | KY038007 |
| *Cnemaspis kallima* | AA 82 | KY037970 |
| *Cnemaspis kandiana* | AMB 7508 | JX041325 |
| *Cnemaspis pava* | WHT 7261 | KY037979 |
| *Cnemaspis kendallii* | LSHC 6562 | JX041326 |
| *Cryptactites peringueyi* | WC-5738/ PEM R24285 | OM885017 |
| *Cryptactites peringueyi* | JB 21 | KF666814 |
| *Cryptactites peringueyi* | CAS:HERP:186374 | JX041339 |
| *Cyrtodactylus angularis* | FMNH HERP265815 | JX041340 |
| *Cyrtodactylus annandalei* | CAS 215722 | JX440524 |
| *Cyrtodactylus oldhami* | JB 126 | JX440548 |
| *Cyrtodactylus irregularis* | FMNH HERP258697 | JX041341 |
| *Cyrtodactylus jarujini* | FMNH HERP255472 | JX041342 |
| *Cyrtodactylus philippinicus* | FMNH HERP236073 | JX041344 |
| *Cyrtopodion rohtasfortai* | PMNH 2391 | KC151979 |
| *Cyrtopodion kohsulaimanai* | PMNH 2388 | KC151965 |
| *Cyrtopodion scabrum* | TG00 109 | JX041345 |
| *Dixonius aaronbaueri* | ZFMK 87274 | HM997152 |
| *Dixonius melanostictus* | a2 | HM997153 |
| *Dixonius siamensis* | LLG 7328 | EU054299 |
| *Dixonius vietnamensis* | FMNH HERP263003 | EU054297 |
| *Ebenavia inunguis* | ZCMV 2099 | JX041348 |
| *Ebenavia inunguis* | ZSM 81/2005 | EF536191 |
| *Elasmodactylus tuberculosus* | JB 85 | KY224214 |
| *Elasmodactylus tetensis* | PEM R05551 | JX041349 |
| *Geckolepis maculata* | FGZC 463 | EU054235 |
| *Geckolepis typica* | FGZC 2343 | EU054233 |
| *Gehyra australis* | AMS 139934 | JN019081 |
| *Gehyra brevipalmata* | USNM 559786 | JN393910 |
| *Gehyra dubia* | AMS 152245 | JN393911 |
| *Gekko mindorensis* | KU 303912 | JN019076 |
| *Gekko monarchus* | PEM R5412 | JN019077 |
| *Gekko kuhli* | RMB 1134 | JX041423 |
| *Gekko lionotum* | CAS HERP221168 | JX515627 |
| *Goggia braacki* | AMB 4265 | KM073689 |
| *Goggia essexi* | LSUMZ H1655 | MF154676 |
| *Goggia lineata* | AMB 4762 | JX041353 |
| *Hemidactylus greefii* | CAS 219044 | EU268369 |
| *Hemidactylus angulatus* | MVZ 245438 | EU268367 |
| *Hemidactylus longicephalus* | CAS 218939 | HM559637 |
| *Hemiphyllodactylus aurantiacus* | AMB (no number) | JN393933 |
| *Hemiphyllodactylus titiwangsaensis* | LSHC 7208 | JN393934 |
| *Hemiphyllodactylus yunnanensis* | FMNH 258695 | JN393935 |
| *Heteronotia planiceps* | AMS 140331 | EU054300 |
| *Heteronotia binoei* | AMS 151170 | EU054301 |
| *Homopholis arnoldi* | MCZ R190441 | KF982287 |
| *Homopholis mulleri* | MCZF 38772 | EU054169 |
| *Homopholis walbergii* | AMB 8123 | EU054248 |
| *Lygodactylus bivittis* | FGMV 2001/A21 | JX041380 |
| *Lygodactylus bradfieldi* | AMB 7628 | JX041381 |
| *Lygodactylus mirabilis* | FGMV 2000/B3 | JX041382 |
| *Lygodactylus tolampyae* | FGMV 2001/C4 | JX041383 |
| *Lepidodactylus aureolineatus* | ABTC 50554 | MG780702 |
| *Lepidodactylus euaensis* | USNM 322126 | JX515611 |
| *Lepidodactylus lugubris* | AMB 4111 | JX041377 |
| *Luperosaurus angliit* | KU 322189 | JQ437903 |
| *Luperosaurus cumingii* | RMB 3546 | JX041379 |
| *Luperosaurus macgregori* | KU 304850 | JX515624 |
| *Matoatoa brevipes* | FGMV 2002/2237 | EF490777 |
| *Matoatoa brevipes* | JB 86 | KF666816 |
| *Matoatoa brevipes* | JB 221 | KF666815 |
| *Mediodactylus russowii* | AMB 8701 | JX041384 |
| *Mediodactylus spinicauda* | CAS HERP228709 | KC151968 |
| *Mediodactylus brachikolon* | PMNH 2165 | KC151981 |
| *Microgecko persicus* | JB5 | KC151982 |
| *Nactus acutus* | BPBM 20755 | EU054289 |
| *Nactus cheverti* | USNM 561874 | HM997154 |
| *Nactus eboracensis* | USNM 561880 | HM997156 |
| *Nactus vankampeni* | FK 11384 | EU054295 |
| *Narudasia festiva* | AMB 3243 | JX041387 |
| *Narudasia festiva* | FB 501 | OM897217 |
| *Narudasia festiva* | FB 503 | OM897218 |
| *Ramigekko swartbergensis* | JB 47 | JX041305 |
| *Ramigekko swartbergensis* | MC(VC111) | KF666799 |
| *Ramigekko swartbergensis* | AMB 8713 | KF666793 |
| *Ramigekko swartbergensis* | JB 48 | KF666796 |
| *Ramigekko swartbergensis* | JB 33 | KF666795 |
| *Ramigekko swartbergensis* | MC(ASK53) | KF666818 |
| *Ramigekko swartbergensis* | MC(MPU041) | KF666798 |
| *Ramigekko swartbergensis* | MC(MPU039) | KF666797 |
| *Ramigekko swartbergensis* | JB124 | KF666794 |
| *Pachydactylus kochii* | CAS HERP214308 | JX041336 |
| *Pachydactylus wahlbergii* | NMZ 16974 | JX041337 |
| *Pachydactylus punctatus* | PEM R12461 | KY224233 |
| *Pachydactylus waterbergensis* | MCZ R184751 | KY224241 |
| *Pachydactylus rugosus* | CAS HERP201905 | JX041395 |
| *Pachydactylus weberi* | PEM R12449 | HQ165960 |
| *Pachydactylus rangei* | MCZ R83725 | JX041394 |
| *Paroedura picta* | ZSM 85/2005 | EF536196 |
| *Paroedura androyensis* | ZCMV 2483 | EF490774 |
| *Paroedura bastardi* | ZSM 180/2004 | EF536211 |
| *Paragehyra gabriellae* | FGZC 2366 | JX041399 |
| *Perochirus ateles* | DWB | JN393938 |
| *Phelsuma inexpectata* | JB 56 | JN393939 |
| *Phelsuma laticauda* | FGZC 2705 | JX041401 |
| *Pseudogekko compressicorpus* | KU 324426 | JQ437898 |
| *Pseudogekko brevipes* | RMB 3282 | KF875325 |
| *Pseudogekko smaragdinus* | KU H303995 | JX041420 |
| *Ptenopus carpi* | CAS HERP214534 | JX041422 |
| *Rhoptropus bradfieldi* | MCZ R183736 | JX041432 |
| *Rhoptropus afer* | MCZ R183711 | JX041430 |
| *Stenodactylus doriae* | JB 20 | KC151985 |
| *Tropiocolotes nubicus* | JB 123 | KC151991 |
| *Tropiocolotes tripolitanus* | MVZ HERP238922 | JX041459 |
| *Urocotyledon inexpectata* | MCZ F38723 | JX041461 |
| *Uroplatus alluaudi* | ZSM 251/2004 | EF490793 |
| *Uroplatus ebenaui* | ZSM 322/2004 | EF490786 |
| *Uroplatus guentheri* | ZSM 476/2001 | EF490778 |
| *Uroplatus giganteus* | ZSM 55/2005 | EF490790 |
| **Outgroup** |  |  |
| *Phyllodactylus tuberculosus* | KU 289758 | JX041411 |
| *Phyllodactylus delcampoi* | JAC 21928 | JX041407 |
| *Phyllodactylus bugastrolepis* | ROM 38489 | JX041406 |
| *Phyllodactylus bordai* | AMCC 118242 | JX041405 |
