# Supplementary material for: ﻿A new species of feather-tailed leaf-toed gecko, Kolekanos Heinicke, Daza, Greenbaum, Jackman, Bauer, 2014 (Squamata, Gekkonidae) from the poorly explored savannah of western Angola
Source: Zookeys. 2022 Nov 2;1127:91–116. doi: 10.3897/zookeys.1127.84942 (PMC9836571; doi:10.3897/zookeys.1127.84942)
Supplement: Supplementary material 2 — Results of the analysis of morphometric differences between Kolekanos spp. and sexes [file zookeys-1127-091_article-84942__-s002.docx]

|  | **Species** | | **Sex** | |
| --- | --- | --- | --- | --- |
|  | **F-value** | **p-value** | **F-value** | **p-value** |
| **SVL*** | 2.676 | 0.124 | 0.552 | 0.485 |
| **TrunkL** | 1.668 | 0.219 | 0.615 | 0.463 |
| **HL** | 0.350 | 0.564 | 0 | 1 |
| **HW** | 59.451 | **0.000** | 0.2 | 0.670 |
| **HH** | 2.158 | 0.166 | 0.111 | 0.750 |
| **OD** | 1.243 | 0.285 | 0.429 | 0.537 |
| **EL** | 0.474 | 0.503 | 0.655 | 0.537 |
| **CL** | 0.463 | 0.508 | 1 | 0.356 |
| **FL** | 7.764 | **0.015** | 1.421 | 0.278 |
| **NE** | 4.371 | 0.057 | 1 | 0.356 |
| **SE** | 5.905 | **0.030** | 0 | 1 |
| **EE** | 0.088 | 0.771 | 3.857 | 0.097 |
| **IN** | 3.843 | 0.072 | 0 | 1 |
| **OO** | 25.834 | **0.000** | 0.158 | 0.705 |
