# Supplementary material for: ﻿A new species of feather-tailed leaf-toed gecko, Kolekanos Heinicke, Daza, Greenbaum, Jackman, Bauer, 2014 (Squamata, Gekkonidae) from the poorly explored savannah of western Angola
Source: Zookeys. 2022 Nov 2;1127:91–116. doi: 10.3897/zookeys.1127.84942 (PMC9836571; doi:10.3897/zookeys.1127.84942)
Supplement: Supplementary material 3 — Principal Component Analysis (PCA) loadings for each morphological variable measured in of Kolekanos spp., including standard deviation (SD), percentage of variance (% Variance) and cumulative proportion for each component. [file zookeys-1127-091_article-84942__-s003.docx]

**Table S3a.** Principal Component Analysis (PCA) loadings for each morphological variable measured in of *Kolekanos* spp., including standard deviation (SD), percentage of variance (% Variance) and cumulative proportion for each component. For abbreviations, see Materials and Methods section.

|  | PC1 | PC2 | PC3 | PC4 | PC5 | PC6 | PC7 |
| --- | --- | --- | --- | --- | --- | --- | --- |
| **SVL** | -0.326225390 | 0.08242686 | -0.087814140 | -0.28936747 | 0.204885015 | -0.04455400 | 0.381883004 |
| **TrunkL** | -0.127858895 | 0.06996204 | -0.607437232 | -0.49464499 | -0.051291205 | -0.05025652 | -0.005508888 |
| **HL** | -0.249076155 | -0.42562233 | -0.148994944 | 0.19032393 | 0.099209561 | -0.09071099 | 0.411511086 |
| **HW** | -0.356137612 | 0.11597305 | 0.001450894 | 0.10267435 | -0.065009186 | 0.30886332 | -0.210755419 |
| **HH** | -0.254114392 | -0.04323206 | -0.027084353 | 0.08801464 | -0.393911702 | -0.78983051 | -0.338368160 |
| **OD** | -0.329805344 | -0.09677273 | 0.056421730 | 0.03042188 | 0.389302606 | -0.05354714 | -0.121775536 |
| **EL** | -0.005707639 | -0.52411433 | 0.439064163 | -0.21786628 | 0.067090939 | 0.09816531 | -0.308268055 |
| **CL** | -0.207377954 | -0.12425554 | 0.465825250 | -0.36055480 | -0.361726242 | -0.04492683 | 0.438732323 |
| **FL** | 0.092823420 | -0.55712200 | -0.329793355 | -0.14834084 | -0.003585043 | 0.16867564 | -0.285502276 |
| **NE** | -0.328556981 | -0.12817532 | -0.100950169 | 0.30630493 | 0.282932981 | -0.10104823 | 0.071921611 |
| **SE** | -0.348273524 | -0.11100068 | 0.010319446 | 0.25943915 | -0.042165152 | 0.14421255 | -0.044868497 |
| **EE** | -0.227449662 | 0.21193546 | 0.187379809 | -0.46604059 | 0.392111043 | -0.09029087 | -0.309933348 |
| **IN** | -0.302388450 | -0.05448534 | -0.146530035 | -0.10415269 | -0.502937252 | 0.36346409 | -0.079681567 |
| **IO** | -0.301562327 | 0.32452656 | 0.108518578 | 0.16586751 | -0.103507595 | 0.23193046 | -0.180091597 |
| **SD** | 25.637 | 14.779 | 11.7940 | 11.2583 | 0.96137 | 0.76924 | 0.61374 |
| **%Variance** | 0.4695 | 0.1560 | 0.09936 | 0.09053 | 0.06602 | 0.04227 | 0.02691 |
| **Cumulative Proportion** | 0.4695 | 0.6255 | 0.72484 | 0.81537 | 0.88139 | 0.92365 | 0.95056 |

**Table S3a. [Continuation…]** Principal Component Analysis (PCA) loadings for each morphological variable measured in of *Kolekanos* spp., including standard deviation (SD), percentage of variance (% Variance) and cumulative proportion for each component. For abbreviations, see Materials and Methods section.

|  | PC8 | PC9 | PC10 | PC11 | PC12 | PC13 | PC14 |
| --- | --- | --- | --- | --- | --- | --- | --- |
| **SVL** | -0.41132570 | 0.32019559 | -0.126783926 | 0.32270727 | -0.189257242 | 0.31311001 | -0.28241107 |
| **TrunkL** | 0.41616019 | 0.20075838 | 0.032355652 | -0.19642238 | -0.081426819 | 0.04436535 | 0.31336309 |
| **HL** | -0.21882373 | -0.43528571 | -0.079719641 | -0.12681885 | -0.027552593 | 0.09030572 | 0.49220081 |
| **HW** | -0.14622410 | 0.23256075 | 0.197541860 | 0.41948920 | 0.460459861 | -0.06492265 | 0.44545856 |
| **HH** | -0.12088270 | 0.03323600 | -0.037869199 | 0.08876737 | 0.017366866 | 0.07808715 | -0.01244400 |
| **OD** | 0.53511236 | -0.23583222 | -0.420078151 | 0.23787450 | 0.273456869 | 0.11260252 | -0.21454950 |
| **EL** | 0.07376687 | 0.30434986 | -0.129477239 | 0.05973595 | -0.414539702 | 0.07586218 | 0.28259575 |
| **CL** | 0.17142432 | 0.07591471 | 0.085548472 | -0.22856587 | 0.389252322 | -0.12056409 | -0.09191497 |
| **FL** | -0.30709345 | 0.04948942 | -0.006708698 | -0.20655232 | 0.417065405 | 0.08451691 | -0.34598156 |
| **NE** | 0.01707091 | 0.39238839 | 0.048217473 | -0.22878870 | -0.108356415 | -0.65947984 | -0.14348930 |
| **SE** | 0.24874686 | -0.03677898 | 0.645720538 | -0.12126580 | -0.203645182 | 0.42740923 | -0.23525048 |
| **EE** | -0.26621523 | -0.40925599 | 0.289369680 | -0.18306991 | -0.033951351 | -0.18943714 | 0.02081470 |
| **IN** | -0.02311098 | -0.34727499 | -0.179802641 | 0.22163033 | -0.343520461 | -0.33873628 | -0.21573354 |
| **IO** | -0.15714124 | 0.11242076 | -0.447358434 | -0.59380239 | -0.003621055 | 0.27054011 | 0.06517755 |
| **SD** | 0.50541 | 0.36401 | 0.3470 | 0.30468 | 0.22789 | 0.17381 | 0.09400 |
| **%Variance** | 0.01825 | 0.00946 | 0.0086 | 0.00663 | 0.00371 | 0.00216 | 0.00063 |
| **Cumulative Proportion** | 0.96881 | 0.97827 | 0.9869 | 0.99350 | 0.99721 | 0.99937 | 100.000 |
